# Genetic mapping and nuclear interactions of an incompatibility response in *Agaricus bisporus*

**DOI:** 10.1093/g3journal/jkag152

**Published:** 2026-06-20

**Authors:** Karin Scholtmeijer, Ben Auxier, Patrick Hendrickx, Brian Lavrijssen, Alfons J M Debets, Johan J P Baars, Duur K Aanen, Arend F van Peer

**Affiliations:** Mushroom Breeding Group, Wageningen University and Research, Wageningen 6700HB, the Netherlands; Laboratory of Genetics, Wageningen University and Research, Wageningen 6700HB, the Netherlands; Mushroom Breeding Group, Wageningen University and Research, Wageningen 6700HB, the Netherlands; Mushroom Breeding Group, Wageningen University and Research, Wageningen 6700HB, the Netherlands; Laboratory of Genetics, Wageningen University and Research, Wageningen 6700HB, the Netherlands; Mushroom Breeding Group, Wageningen University and Research, Wageningen 6700HB, the Netherlands; Laboratory of Genetics, Wageningen University and Research, Wageningen 6700HB, the Netherlands; Mushroom Breeding Group, Wageningen University and Research, Wageningen 6700HB, the Netherlands

**Keywords:** button mushroom, fusion, QTL analysis, heterokaryon incompatibility

## Abstract

During cultivation, mixing of different heterokaryotic individuals of the button mushroom, *Agaricus bisporus*, generally reduces yield. This phenomenon could be caused by direct antagonistic responses and/or reduced synchronization by not forming a chimeric hyphal network. In other fungi, highly divergent alleles for a set of genes affect successful network formation between individuals either by preventing fusion or, more commonly, triggering cell death post-fusion. To understand this process in *A. bisporus*, it is important to identify the allelic variants allowing these fungi to discriminate self from nonself. Leveraging a recently described cell death staining method utilizing Evans Blue to visualize mycelial compatibility, we provide results of a first genetic mapping of incompatibility alleles in *A. bisporus*. Crossing strains between *A. bisporus* var. *bisporus* and *A. bisporus* var. *burnetti* we find segregation ratios of compatible progeny generally consistent with 3 nuclear loci. To identify these regions, we first use a set of single chromosome substitution lines (CSLs), produced by genotyping progeny with recombination skewed to the very chromosome ends. We localize the main effect to be between 2 and 3 chromosomes, depending on the common nucleus of interacting heterokaryons. Using genome-wide markers for 167 sexual progeny, we identify loci controlling mycelial compatibility on chromosomes 4, 6, and 7, the same chromosomes as indicated by CSLs. Notably, while the choice of a common nucleus seemed to affect the compatibility of CSLs, it does not seem to affect the loci identified in the sexual progeny. The ability to mix different strains of this mushroom-forming fungus could allow additional cultivation approaches, combining strains with complementary characteristics. These results provide a starting point toward understanding the molecular mechanisms underlying this fundamental property of hyphal networks in basidiomycetes.

## Introduction

The distinction between self and nonself interactions in fungi is fundamentally important. As the thread-like hyphae of an individual, the mycelium, grow through a substrate, they can encounter hyphae of other individuals. When this occurs between hyphae of the same species, generally the 2 hyphae fuse, and if they are from different individuals this fused cell is rapidly killed (Garnjobst and Wilson 1956). This prevention of fusion between nonself individuals has the benefit of preventing the spread of deleterious elements like nuclei or viruses (Debets and Griffiths 1998; Grum-Grzhimaylo et al. 2021). In Basidiomycetes, the distinctive heterokaryotic state, with 2 distinct haploid genotypes in a common cytoplasm, presents additional vulnerabilities as mating must accommodate the fusion between 2 homokaryotic individuals (Rayner 1991; James 2015; Auxier et al. 2022). This incompatibility is expressed as 2 potentially overlapping phenomena; *mycelial compatibility* where secondary characteristics of mycelia such as metabolite secretion or aerial hyphae produce a zone of interaction aside from hyphal fusion, and *somatic incompatibility* where the fusion of hyphae between 2 individuals leads to cell death in the fusion zone (Worrall 1997).

This cell death is caused by cytoplasmic proteins, whose interaction presumably triggers downstream processes causing death of the fused cell (Saupe et al. 1996). For sustained fusion between 2 hyphae, the 2 cytoplasms must encode the same versions of the respective proteins. This is a version of synthetic lethality, where cell death is triggered by a combination of alleles not seen in nature (Dobzhansky 1946). For sustained fusion between 2 hyphae, the 2 cytoplasms must encode the same versions of the respective proteins. The alleles encoding these proteins form a sort of barcode that must match to allow stable fusion (Gonçalves and Glass 2020). Sustained fusions between hyphae allows for persistent social interactions, functionally defining an individual. The molecular details of this system have been worked out in some detail in Ascomycete fungi, particularly *Neurospora crassa* (Daskalov et al. 2020) and *Podospora anserina* (Clavé et al. 2024) and between 10 and 15 genes each with multiple alleles have been identified. This high number of genes makes it unlikely that unrelated individuals have the same alleles for all loci, thus generally preventing fusion between different individuals and providing an effective non-self recognition system (Nauta and Hoekstra 1994; Gonçalves and Glass 2020). However, the genes responsible for this in Basidiomycetes are unknown.

Previous studies on the genetics of incompatibility in several mushroom-forming species indicates that generally several loci are involved. In *Amylostereum* it was indicated that at least 2 loci segregated between 2 individuals (van der Nest et al. 2008), and in *Heterobasidion annosum* 3 to 4 loci (Hansen et al. 1993; Lind et al. 2007). The wood-rotting *Serpula lacrymans* had 3 loci segregating, although this species seems to have low genetic diversity overall (Kauserud et al. 2004, 2006). In the wood-decay fungus *Phellinus gilvus*, it was found that a single locus was the primary contributor to the phenotype (Rizzo et al. 1995). Those studies suggest that the number of loci regulating incompatibility in Basidiomycetes is lower than in Ascomycetes.

Currently, button mushroom cultivation requires the use of single strains. This is because cultivation of mixed cultures leads to dramatic reductions in yield (Kerrigan and Wach 2014; Scholtmeijer et al. 2025). This incompatibility response between heterokaryons has also been demonstrated to prevent virus transmission (O’Connor et al. 2021). In theory, knowing the genes, or at least narrow genetic regions, causing incompatibility could allow selection of individuals that would be somatically compatible. Availability of somatically compatible, but genetically diverse, strains could allow selection on more specialized aspects of cultivation.

Here, we present results from mapping the incompatibility loci in *Agaricus bisporus*. Using a set of single chromosome substitution lines (CSLs) as well as sexual *A. bisporus* var*. bisporus* X *A. bisporus* var. *burnetti* populations of meiotic progeny, we demonstrate the segregation of 3 loci. These loci were genetically mapped but could not be resolved to a gene level. To understand the interaction between nuclei in a heterokaryon, we leveraged the multiple mating alleles to mate the progeny with different mating partners. Comparisons between these populations showed that the loci segregating seemed to be independent of the partner nucleus, indicating dominant interactions.

## Materials and methods

### Terminology

For clarity, here we use *heterokayon* to refer to a fungal individual with 2 nuclear genotypes, necessary to produce mushrooms. We use *homokaryon* to refer to isolates with a single type of nuclei, which can be derived either from screening the often heterokaryotic sexual spores for rare homokaryotic ones, or from protoplasting to isolate individual genotypes. Two homokaryons can be *mated* to produce a heterokaryon, assuming the mating-type loci are compatible, and if mushrooms are produced leading to sexual spores, we refer to this as *crossing*. Following the terminology of Worrall, we use *common nucleus* when we independently mate different homokaryons with the same common homokaryon, producing related heterokaryons. Finally, we reserve the term *interaction* for testing incompatibility between heterokaryons. In this work, matings occur exclusively between homokaryons, and interactions are exclusively tested between heterokaryons.

### Strains and culture conditions

Our starting material was 2 heterokaryotic *A. bisporus* strains, 2-spored A15 and 4-spored Bisp015 (Table 1). We recovered the 2 homokaryotic genotypes of each heterokaryon by protoplasting (Sonnenberg et al. 1988), using PCR with mating locus allele specific primers to identify the 2 component nuclei. The 2 recovered homokaryotic components of A15 we designated as A and B, and the 2 from Bisp015 (Kerrigan 1995, 1996) as C and D. These strains have compatible mating types in all 6 pairwise combinations. To produce CSLs (see below), we used 2 homokaryons, H39 (clonally related to A) and H97 (clonally related to B). The strains listed and genetic codes are found in Table 1. Note that we hereafter refer to E as A and F as B, due to the clonal relationship. For routine culture, cultures were grown on MMP Agar (Scholtmeijer et al. 2025) at 24 °C.

**Table 1.** *A. bisporus* strains, all with compatible mating types, used in genetic crosses.

Homokaryotic strain	Source	Species
A	A15 protoplasting	*A. bisporus* var. *bisporus*
B	A15 protoplasting	*A. bisporus var. bisporus*
C	Bisp015 protoplasting	*A. bisporus* var. *burnetti*
D	Bisp015 protoplasting	*A. bisporus* var. *burnetti*
E	H39 (clonally related to A)	*A. bisporus* var. *bisporus*
F	H97 (clonally related to B)	*A. bisporus* var. *bisporus*

### Chromosome substitution lines

To identify which chromosome(s) harbor alleles for the incompatibility interaction, we produced a set of single CSLs. For this, we crossed E and F, and screened basidiospores for colonies with a single mating type, ie homokaryons (see below). Using 42 SNP markers spread across the chromosomes, we genotyped the single-spore isolates (SSI) to recover isolates with essentially non-recombined chromosomes, which is possible as recombination in *A. bisporus* var. *bisporus* predominantly occurs at the chromosome ends (Sonnenberg et al. 2016) and with the genetic background of H39 except for 1 of the 13 chromosomes of H97. We produced one of each CSL except for chromosomes 4 and 7, for which we recovered 2 independent CSLs.

These homokaryotic CSL isolates were then mated with either C or D homokaryons, and interactions with AC or Ad were measured using Evans Blue staining as described previously (Scholtmeijer et al. 2025). We use this as a measure of incompatibility, as this stain is excluded from living tissue and thus highlights dead hyphae.

### Meiotic progeny

To produce recombinant meiotic progeny, we crossed B and D, collecting SSI from spore prints from multiple mushrooms. These SSI were screened for homokaryotic progeny using primers designed for the specific allele of the HD mating-type locus (Supplementary Tables 1 and 2). These meiotic progeny were mated with either A or C, and incompatibility interactions were also scored as for the CSLs. In addition, we produced a second meiotic population by crossing A with C. These were treated as above, but mated instead with either B or D.

### Genetic mapping

For the population resulting from the cross of B with D, we genotyped in two differing ways. First, we genotyped 96 homokaryotic SSI with 106 SNP markers using a competitive allele specific PCR (KASP assay). To this population, we added 71 additional isolates, which were processed for whole genome sequencing with paired-end 150 bp short reads. Reads were aligned to the *A. bisporus* H97 version 3.1 reference genome (Sonnenberg et al. 2016) with bwa-mem v.0.7.17 (Li, 2013) using the default settings and variants were called with FreeBayes v1.1.0 (Garrison and Marth 2012) using default settings. The resulting variant file was filtered to retain only variants of type SNP with a minimum read depth of 10, and the filtered variant file was converted in a genotype file using dedicated scripts. The genotype at the same 106 SNP loci was determined and merged into 1 genotype file.

Genetic mapping was performed using the R/qtl2 package (Broman et al. 2019). We removed any individuals with >25 markers with missing genotypes and removed potential clones. We removed 1 marker, Ch5_6859, that showed segregation distortion (0.86 frequency). After filtering, the dataset consisted of 96 markers and 147 individuals. We constructed a linkage map using the minimum spanning tree approach implemented in ASMap (Wu et al. 2008; Taylor and Butler 2017), resulting in 23 linkage groups using a 1 × 10^−4^ significance threshold. We manually merged the 23 linkage groups into the 13 described chromosomes (Sonnenberg et al. 2020). We visualized the resulting genetic map with LinkageMapView (Ouellette et al. 2018).

We mapped the incompatibility trait using Haley–Knott regression using the binary model as our phenotypes are binary. We determined significance thresholds using 1,000 permutations of the data, using α = 0.05. Significantly associated windows of the genome were based on the peak LOD value bordered by a drop of 1 in LOD from this peak on either side.

## Results

### Genetic basis for incompatibility between *A. bisporus* isolates

**Fig. 1.**
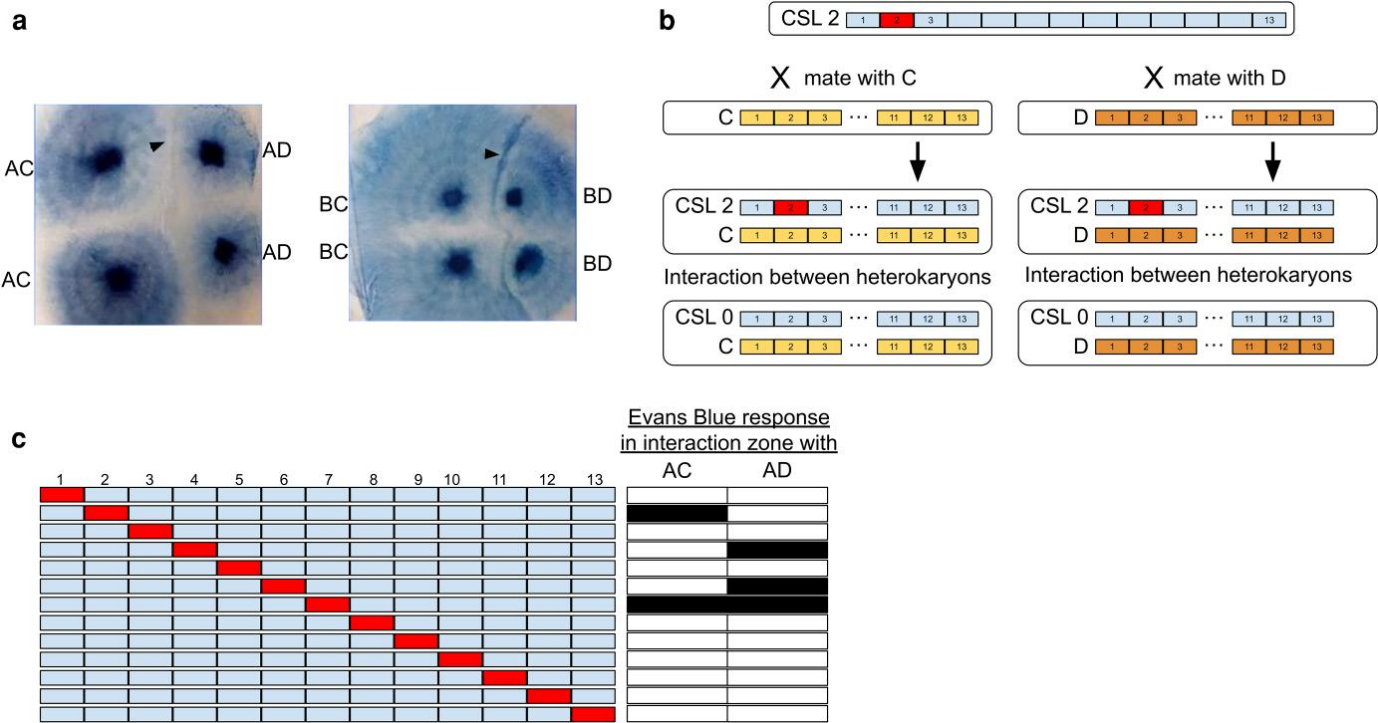
Incompatibility assessed between *A. bisporus* strains A and B, when mated with C or D isolates. a) Incompatible response under Evans Blue staining, with a dark blue line forming between the different heterokaryons (arrowheads) but not between self-interaction controls. b) Construction of the CSLs and subsequent interaction testing. c) Results of interactions scoring for the 15 CSLs tested against AC or Ad heterokaryons. Black boxes indicate incompatible responses due to the corresponding B chromosome in the CSL.

Initial tests showed that heterokaryons we produced displayed an incompatible response, of variable strength, between isolates (Supplementary Fig. 1). This incompatible response was not observed between self-pairing of isolates (Supplementary Fig. 1). For example, when we mated A with C and B with C, interactions between this AC heterokaryon and the Bc heterokaryon produced increased Evans Blue staining in the interaction zone (Fig. 1b, arrowhead), while self-interactions of AC with AC or Bc with Bc did not produce an incompatible response. Similarly, when A or B were mated with the common nucleus D, the interactions between Ad and BD heterokaryons produced an incompatible response, while self-interactions did not (Fig. 1b).

To determine the chromosomes associated with this response, we produced a set of single CSLs (Fig. 1a), a set of lines with 12 chromosomes of A and a single non-recombinant chromosome from B. Genotyping across 42 markers, we identified strains that were essentially the genotype of A, but with 1 B chromosome (Supplementary File 1). We recovered a single CSL for each chromosome, with duplicates for chromosome 3 and 4. By mating these 15 CSLs with either homokaryon C or D, we produced 2 sets of heterokaryons. These 2 sets of heterokaryons were tested for incompatibility against the AC heterokaryon (common nucleus C) or Ad (common nucleus D) using Evans Blue staining to detect cell death in the interactions zone (Supplementary Fig. 2). When mated to the common nucleus C, among the set of CSLs those with chromosome 2 and 7 from B showed incompatibility to AC (Fig. 1c; Supplementary Fig. 2). When mated with common nucleus D, CSL-4, 6, and 7 showed incompatibility with Ad (Fig. 1c; Supplementary Fig. 3). For the duplicate CSLs for chromosome 3 and 4, the results were consistent (Supplementary Fig. 3). Chromosome 1, which contains the mating type locus, showed no involvement in incompatibility.

### Segregation of incompatibility within recombinant populations

From the B/D population, we attempted to mate all 92 progeny with both A and C. When mated with strain A, 14.3% of B/D offspring were compatible with AB, and 9.4% with Ad. We observed that 16 individuals (10.9%) did not successfully mate with strain A. When we instead mated the 92 progeny with isolate C, we found that 14.6% were compatible with the Bc heterokaryon, while only 5.4% were compatible with the CD heterokaryon.

We also crossed A with D, collecting 98 homokaryotic SSIs from the A/D population, which were mated with either strain B or C. When mated with B, 11% of this A/D population was compatible in interactions with an AB dikaryon (Table 2; top row). Likewise, 12% of the A/D population was, when mated with C, compatible with the CD heterokaryon (Table 2; fourth row). Both values were statistically consistent with 3 locus segregation, with a *P* > 0.05 from an exact binomial test.

**Table 2.** Segregation of compatible reactions in hybrid progeny pools.

Set^a^	Pop.	Mating partner	Interact. individ.	Interaction notation^b^	Segregation testing	Compatible frequency^c^	Three loci hypothesis^d^
	(**A/D**)	B	AB	(A/D + B) vs (A + B)	D different from A	11% (98)	*P* = 0.87
			BD	N/D	N/D	N/D	N/D
		C	AC	N/D	N/D	N/D	N/D
			CD	(A/D + C) vs (C + D)	A different from D	12% (98)	*P* = 1.00
1	(**B/D**)	A	AB	(B/D + A) vs (A + B)	D different from B	14.3% (84)	*P* = 0.61
2			Ad	(B/D + A) vs (A + D)	B different from D	9.4% (75)	*P* = 0.48
3		C	b c	(B/D + C) vs (B + C)	D different from B	14.6% (82)	*P* = 0.50
4			CD	(B/D + C) vs (C + D)	B different from D	5.4% (92)	*P* = 0.04

^a^Used for QTL mapping in Fig. 3.

^b^Underlined letters indicate shared genotypes between the 2 heterokaryons, note N/D = not done.

^c^Numbers in brackets indicate total number of progeny that were used for phenotyping.

^d^ *P*-values represent exact binomial test assuming segregation of compatible progeny at 0.125.

### Genetic map of the *A. bisporus* var. *bisporus* × *A. bisporus* var. *burnetti* BD cross

**Fig. 2.**
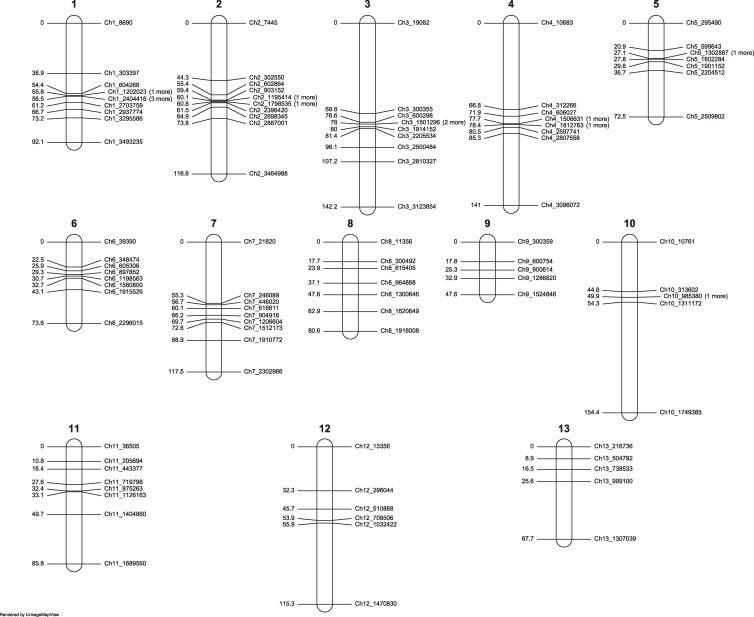
Genetic map of meiotic progeny of A (*A. bisporus* var. *bisporus*) × D (*A. bisporus* var. *burnetti*). Spacing of the 108 markers across the 13 chromosomes is depicted, scaled along the chromosomes based on cM distance (left side). Physical position is found in the marker name, with markers being spaced ∼0.3 Mb apart.

To increase resolution of genetic mapping, we crossed B with genotype D, a homokaryon from the closely related *A. bisporus* var*. burnetti*, a cross described to have an increased frequency of non-telomeric crossovers (Foulongne-Oriol et al. 2010). Sequencing of these meiotic progeny showed a total genetic distance of 1,326.8 cM spread across the 13 chromosomes (Fig. 2). Particularly for the larger chromosomes, despite even large physical distances between markers, genetic distances were highly skewed, with large portions of chromosomes 2, 3, 4, and 7 being essentially non-recombinant.

### Mapping of incompatibility within the (B/D) population

The Evans Blue phenotype across the offspring was variable, with some isolates producing less staining overall (compare left and right isolates in Supplementary Fig. 4). Compatible interactions generally had less staining than the mycelia near the inoculation site (Supplementary Fig. 4a to d). When incompatibility was observed, as a dark blue line between the genotypes, it varied between a weak interaction zone (Supplementary Fig. 4e and f), to a darkly pigmented zone (Supplementary Fig. 4g and h). For the subsequent genetic mapping, we split the interactions into either compatible or incompatible, lumping all gradients of weakly/strongly incompatible into 2 categories.

**Fig. 3.**
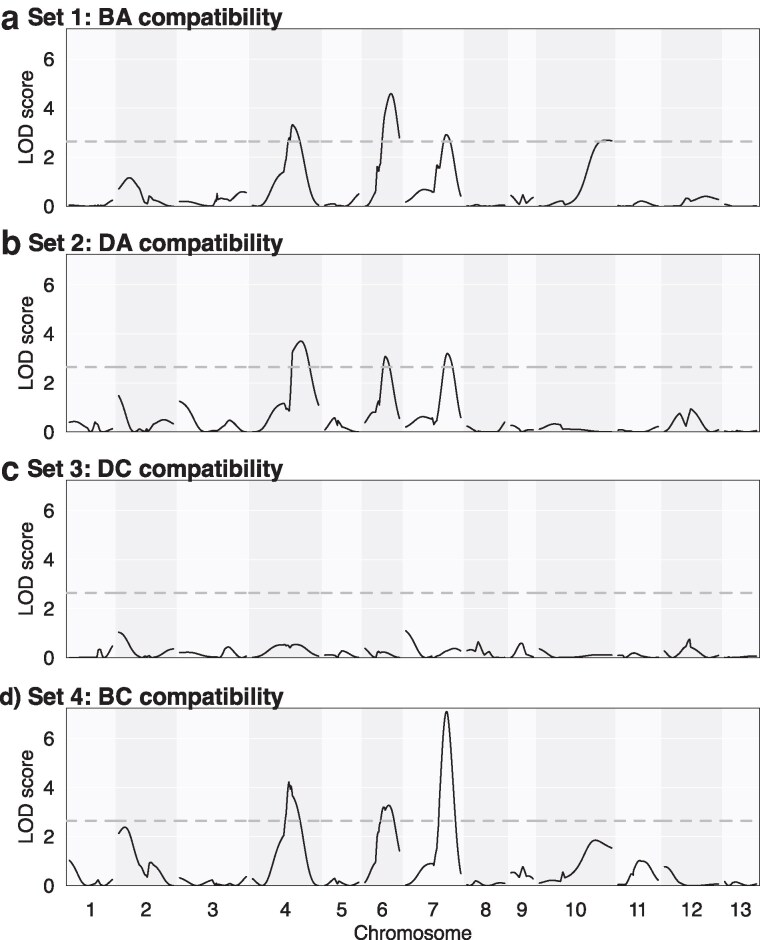
QTL mapping of compatibility within the B/D population. a and b) Interactions of B/D progeny when mated with A and interacted against either BA or DA heterokaryon. c and d) Results when the same population was mated with C and interacted with DC or Bc. Different chromosomes are indicated by differential vertical shading. Statistical significance is indicated by horizontal dotted line, and was determined by a permutation test.

We phenotyped the interaction of the B/D population against 4 different heterokaryons (Table 2). Using genetic information from 96 markers spread across the 13 chromosomes, we mapped the loci allowing compatibility. QTL mapping results in peaks on chromosome 4, 6, and 7 being found in the AB, Ad, and Bc interactions (Fig. 3). A significant peak was also found on chromosome 10 for interactions involving the AB individuals. Interactions involving the CD individual did not result in any significant peaks.

## Discussion

Our results show that several loci contribute to the incompatibility phenotype of *A. bisporus*, spread across multiple chromosomes. In the meiotic progeny, genetic mapping showed a consistent association on chromosomes 4, 6, and 7. Across the 4 common nuclear backgrounds tested, we recovered peaks centered at similar regions of the chromosomes. This likely indicates that the effect of the common nucleus is not strong in this experiment. In interactions between offspring of B and D when mated with C, the compatibility ratio compared with DC segregated significantly different from a 3-locus model, although such a marginally significant result is not unexpected when testing across multiple backgrounds.

Our use of CSLs allowed for rapid screening of the incompatibility phenotype, and likely would be of use for screening other dominant phenotypes. Such segregating populations are used in species like *Aspergillus niger*, as well as plants like *Arabidopsis thaliana* among others (Debets et al. 1993; Wijnen et al. 2024). One caveat of the lines used here is that the CSLs are not composed of entire non-recombinant chromosomes, as there is telomeric recombination. While it is possible that some of the nonself recognition genes are located near telomeres, as seen in *N. crassa* (Wang et al. 2023), this is not likely in our experiment. The meiotic crossing data showed that the identified QTL were localized to the center of the chromosomes.

In the model Ascomycete, *N. crassa*, it was noted early on that the mating type was involved in nonself recognition (Garnjobst 1953). The molecular details of this interaction have been determined (Jacobson 1992; Shiu and Glass 1999), and it is often thought that this is a common factor in fungal nonself recognition. Here, we do not find any association between the chromosome where the functional mating locus resides, chromosome 1, and incompatibility. This lack of association may be more common, as even in most Ascomycetes it is not found (Choi et al. 2012; Auxier et al. 2024). These data are consistent with our previous model, where the rapid nuclear migration with compatible mating types may allow physical “escape” from the incompatibility response, and not transcriptional regulation (Auxier et al. 2021).

It has been suggested previously that using an experimental design where a genotype is shared across heterokaryons, a so-called common nucleus, can simplify the genetic analysis (Worrall 1997). Our experiments indicate this may not be a safe assumption, as the comparison between set 2 and 4 show significantly different segregation ratios. We observed that when paired with nucleus A many more offspring are compatible with D than when paired with nucleus C. It may be that the influence of the common nucleus is shielding the effects of an incompatibility locus, something we have speculated on previously (Auxier et al. 2021). Previous studies have made use of a common nucleus when mapping this trait (Hansen et al. 1993), but few have tested the effect of different common nuclei.

Combining the evidence of CSLs and meiotic progeny, we predict strong incompatibility alleles on chromosomes 4, 6, and 7. Finding 3 loci segregating between 2 haploid strains is consistent with findings from other Basidiomycetes like *H. annosum* (Lind et al. 2007). We have also shown that segregation of the nonself recognition response was consistent with 3 loci in *Coprinopsis cinerea*, and that 1 locus contains components resembling nucleotide binding leucine-rich repeats genes (Auxier et al. in prep.). However, whatever the ultimate mechanism, it is important to recognize this is significantly fewer than the number of loci that segregate in Ascomycete species (Gonçalves and Glass 2020). As we have suggested previously, this may be due to the combination of two genomes inside a heterokaryotic mycelium (Auxier et al. 2021). If the alleles for these loci act co-dominantly, then a few loci could provide a large number of potential vegetative compatibility groups. Since we observe this phenomena at a macroscopic level, we are assaying millions of hyphal interactions, and potentially incompatibility could results from hyphae that have become homokaryotic. This seems unlikely because *A. bisporus* hyphae consistently have between 6 and 8 nuclei per compartment near the growing edge (Evans 1959), and 3 to 5 per compartment in older hyphae (Kamzolkina et al. 2006). Additionally, spontaneous decomposition of *A. bisporus* cultures into homokaryotic sectors, the outcome of such a process, is not something that we have observed during cultivation.

Bioinformatic methods to identify nonself recognition genes in Basidiomycetes have produced mixed results. Since many details of the molecular mechanisms of nonself recognition have been worked out in Ascomycetes, and genomic data is rapidly accumulating, large-scale surveys have attempted to transfer this knowledge to mushroom-forming fungi. An initial survey found Basidiomycete homologs for many of the genes used for this process in Ascomycetes. More detailed surveys of the NLR-based mechanisms also found many copies in Basidiomycete fungi (Dyrka et al. 2014). Recent updates in both genomic data and known mechanisms have increased the number of genes found, and highlighted that Basidiomycetes have particularly diverse NLR genes that require additional functional validation (Wojciechowski et al. 2022). However, these studies are limited by the fact that many NLR genes in Ascomycetes are involved in processes other than nonself recognition (Uehling et al. 2017), and so sequence homology has limited power to infer function.

The commercial cultivation of *A. bisporus* means that somatic incompatibility is particularly relevant. Mixing of different genotypes can lead to dramatic yield reductions (Scholtmeijer et al. 2025). In effect, this adds a restriction to cultivation, since all members of the production chain must agree on a single clone to use. A more complete understanding of the incompatibility response may allow us to bypass this requirement. Additionally, one of the major diseases encountered in mushroom cultivation is Mushroom Virus X, which can drastically reduce yields (Ross et al. 1987). This virus consists of a cluster of ss(+)RNAs and is transmitted through hyphal fusion (Gandy 1960; Grogan et al. 2003; O’Connor et al. 2021), and the incompatible response between individuals presumably limits virus transfer, although in laboratory conditions this can be broken through (O’Connor et al. 2021). The mechanism by which viruses can spread despite this cell death is unclear, but may be related to idiosyncratic interactions between virus types and specific incompatibility loci (Choi et al. 2012). With identification of these 3 loci, even in the absence of molecular characterization, it would be possible to test the contribution of these loci in controlling virus transmission.

## Conclusion

This work confirms that there is a genetic basis for incompatibility in *A. bisporus*, with a similar number of loci compared with other Basidiomycetes. Given the importance of somatic incompatibility at a basic biological level, as well as the industrial relevance, understanding this trait is an important future goal. While the specific genes underlying this trait remain unknown in this species, this work provides a path toward unraveling this genetic trait.

## Supplementary Material

jkag152_Supplementary_Data

## Data Availability

All genotype data and code for analyses are available in the publicly available repository https://github.com/BenAuxier/Agaricus_mapping. Recombinant progeny are available from AvP upon request. Sequencing data of the BxD progeny can be found under NCBI accession ID PRJEB98569. Parents B (A15-p2) and D (Bisp015-p2) can likewise be found as SAMEA120298304 and SAMEA120298305, respectively. Supplemental material available at G3 online.
